# Modification of Sunflower Stalks as a Template for Biochar Adsorbent for Effective Cu(II) Containing Wastewater Treatment

**DOI:** 10.3390/ma18245604

**Published:** 2025-12-13

**Authors:** Ruiqi Yang, Xuejian Zhou, Chunhui Zhang, Xinyue Zhang, Qiyu Bao, Yanou Qi, Xiangshi Liu, Mingyuan Sun, Xifeng Lv, Di Cai

**Affiliations:** 1 Key Laboratory of Modern Agricultural Engineering, College of Chemistry and Chemical Engineering, Tarim University, Alar 843300, China; 17615349985@163.com (R.Y.);; 2 College of Life Science and Technology, Beijing University of Chemical Technology, Beijing 100029, China; 3 State Key Laboratory of Green Manufacturing, Beijing University of Chemical Technology, Beijing 100029, China; 4 National Energy R&D Center for Biorefinery, Beijing University of Chemical Technology, Beijing 100029, China

**Keywords:** biochar, activation, chitosan, Cu(II), adsorption

## Abstract

Sunflower stalks derived biochars were fabricated through sequential alkali/enzymatic pretreatment, carbonization, and chitosan modification, and were used as eco-friendly adsorbents for Cu (II) removal from wastewater. The effects of pH, temperature, adsorption time, and dosage of biochar on Cu (II) adsorption separation from the model solution were comprehensively investigated. Results demonstrated that the chitosan treatment of biochar, obtained from the carbonization of pretreated sunflower straw, significantly altered the porous structure and surface functional groups of the material. Specifically, the biochar carbonized at 500 °C and subsequently treated with chitosan exhibited optimal adsorption performance at pH 5 and 35 °C. Under these conditions, a maximum Cu(II) adsorption capacity of 268.2 mg g^−1^ (of biochar) was realized. Further analysis indicated the Cu(II) adsorption generally followed pseudo-second-order kinetics (*R*^2^ > 0.99). Langmuir isotherm modeling revealed that the biochar modified by NaOH and chitosan displayed the highest correlation coefficient (*R*^2^ > 0.99), suggesting predominantly homogeneous monolayer adsorption. Therefore, the novel low-cost and environmentally friendly biomass-derived adsorbents demonstrate significant potential for effective treatment of the heavy metal-contaminated wastewater.

## 1. Introduction

The rapid expansion of modern industry has intensified concerns regarding the ecological hazards posed by heavy metal contamination [1]. Processes including mining operations, metal smelting, and the disposal of waste power batteries release substantial quantities of toxic Cu(II) [2,3,4]. This has led to escalating pollution in aquatic and terrestrial environments. Consequently, the effective removal of Cu(II) from wastewater represents a significant environmental challenge. Developing cost-effective and eco-friendly Cu(II) management materials is therefore imperative [5]. Biochars, produced via the pyrolysis of waste biomass, offer distinct advantages, including low cost, environmental compatibility, large surface area, and high porosity, which show great promise in the adsorption of toxic heavy metals from contaminated wastewater [6,7]. However, the biochars directly derived from abundant biomasses without any treatment and modification always exhibit inherently low adsorption efficiency and limited capacity for heavy metals [8]. Consequently, strategies to enhance its adsorption performances and heavy metals adsorption capacity through modification warrant further investigation [9].

Chemical modification of biochar using acids (e.g., HCl) or bases (e.g., NaOH) can enhance its adsorption capacity by introducing abundant pore structures and functional groups, thereby creating additional adsorption sites for pollutants [10,11]. However, the effectiveness of chemical modifications is influenced by the types and properties of raw materials. For instance, Su et al. [12] reported a significant decrease in the specific surface area of wheat straw-derived biochar from 4.4 m^2^ g^−1^ to 0.69 m^2^ g^−1^ after KOH pretreatment before carbonization. Conversely, Zhang et al. [13] synthesized three biochars (HCl-BC, KOH-BC, Fe-BCs) from Tieguanyin tea stems using KOH, HCl, and potassium ferrate (K_2_FeO_4_) as activating agents, respectively, followed by investigating the performances for the competitive adsorption of Cu(II) and Zn(II) of the obtained biochars from aqueous solution. The results suggested the Fe-BC650 achieved maximum adsorption capacities of 303.6 mg g^−1^ for Cu(II) and 254.1 mg g^−1^ for Zn(II) in monometallic solutions.

In contrast to chemical modifications, the biological modification of biomass templates primarily using enzymes, such as cellulase, presents another viable approach [14]. The sugars released during cellulase hydrolysis alter the surface properties of the lignocellulosic matrices [15], thereby ultimately enhancing the adsorbent capacity of the corresponding biochars after carbonization. For instance, Li et al. [16] investigated the impact of varying cellulase treatment conditions (cellulase dosage, treatment time, and reaction temperature) on lignocelluloses, focusing on improving the surface characteristics and adsorption performances of the resulting biochar (BC-SR). They found that the BC-SR exhibited high selectivity towards bisphenol A adsorption, and the performance outperformed that of the unmodified biochar in a fixed-bed column, demonstrating superior removal and structural integrity of the cellulase-pretreated lignocellulose-derived BC-SR.

In another research, in contrast to the chemical or biological modification prior to carbonization, Wang et al. [17] synthesized a series of biochars from macadamia nut shells (MNS), followed by modification with carboxymethyl chitosan (CMC) and potassium after carbonization. The received BC-CMC demonstrated the highest adsorption capacity for Zn(II) (97.09 mg g^−1^), attributed to its enhanced abundance of amino, hydroxyl, and carboxyl functional groups after the post-carbonized modification. However, despite extensive studies that have applied chemical and biological modifications of the biochar templates before carbonization, and token chemical modification of the surface functional groups post the carbonization, to our knowledge, studies that combined the front and post modification technologies to synergistically improve the heavy metals adsorption of the biochars still remain limited. In comparison to biomass derived from leaves and roots, stem biomass serves as a more abundant source of biochar. This biomass type exhibits significant adsorption potential, attributed to its substantial volume, high carbon content, and minimal ash production [18].

This study employed sunflower straw (SS), an abundant and high carbon content biomass resource that is recently underutilized as the raw material for biochar adsorbent [19]. After sequential modification by alkali/enzymatic pretreatment, carbonization, and chitosan modification, the Cu(II) adsorption performances of the obtained candidate SS-derived biochars were systematically investigated. The structural properties of the SS-derived biochars were thoroughly analyzed, while the adsorption kinetics and thermodynamics were subjected to fitting analysis, thereby elucidating the reaction mechanism of adsorption. The excellent Cu(II) adsorption performances of the SS-derived adsorbents demonstrate that the sequential modification methods hold broad application prospects in heavy metal adsorption, providing a novel strategy for the high-value utilization of agricultural waste.

## 2. Materials and Methods

### 2.1. Materials

SS was obtained from the local farm of Aral, Xinjiang, China. Cellulase(10,000 U/g), chitosan ((C_6_H_11_NO_4_)_n_, >95% purity), hydrochloric acid (HCl, 37% purity) that were under analytical grade, were purchased from Shanghai Macklin Biochemical Technology Co., Ltd., Shanghai, China. Ethanol (CH_3_CH_2_OH, >95% purity) was purchased from Tianjin Zhiyuan Chemical Reagent Co., Ltd., Tianjin, China. Analytical grade sodium hydroxide (NaOH, >98% purity) was purchased from Tianjin Beilian Fine Chemicals Development Co., Ltd., Tianjin, China, while copper sulfate pentahydrate (CuSO_4_·5H_2_O, >99% purity) was purchased from Tianjin Pharmaceutical Co., Ltd., Tianjin, China.

### 2.2. Preparation of Sunflower Straw Derived Adsorbents

SS was milled into 60 meshes, followed by immersion in 95 vol% ethanol for 6 h [20]. Then, the solids were rinsed repeatedly with distilled water and dried. For the sequential modification of SS for adsorbent production, 3 g of the as-received SS was dispersed in 100 mL of 1 M NaOH solution [21]. The slurry was continuously stirred at 500 rpm for 6 h at 50 °C. Subsequently, the solid was washed with deionized water until reaching neutral pH (~7), followed by drying under vacuum for 12 h at 55 °C. The dried material was subjected to pyrolysis in a tubular furnace under a nitrogen atmosphere and at different temperatures for 2 h (a ramp rate of 5 °C min^−1^). The samples were subjected to carbonization and coded by SS-A-400/500/600 (refer to carbonized temperature). Then, 3 g of the carbonized alkali pretreated SS was added into 100 mL of 10 g L^−1^ chitosan solution and stirred at 500 rpm for 30 min. Subsequently, the solid was washed with deionized water until reaching neutral pH (~7), followed by drying for 24 h at 80 °C. The solids were dried out and coded by SS-AC-400/500/600 (refer to carbonized temperature).

**Figure 1:**
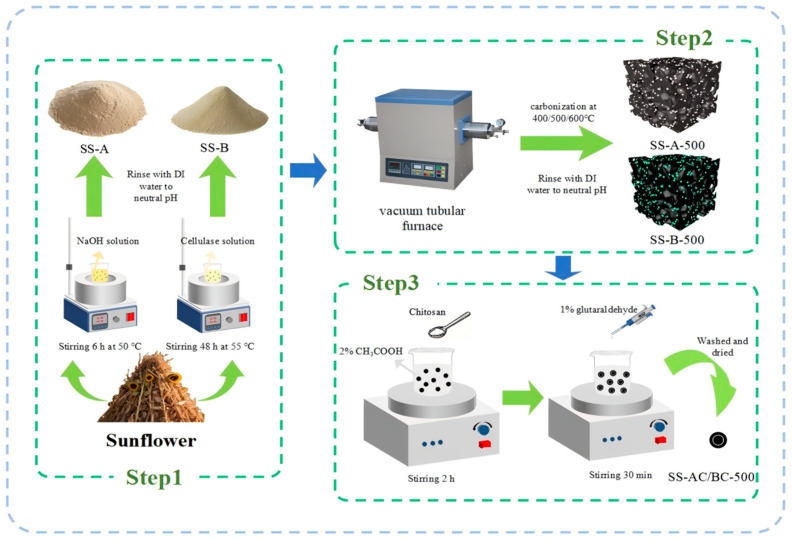
Schematic diagram of the preparation of SS-AC/BC-500 via pyrolysis and activation.

For the sequential enzyme-carbonization-chitosan modification of SS for adsorbent productions, 3 g of pretreated SS was immersed in 100 mL of 2 g L^−1^ cellulase containing buffer (pH was adjusted by citric acid/sodium citrate at ~4.8 [22]). The slurry was reacted at 55 °C and 250 rpm for 48 h. After enzymatic hydrolysis, the obtained solid was washed with distilled water until the surface reached neutral pH (~7). Subsequently, carbonization and the following chitosan modification of the biologically pretreated SS were conducted following the aforementioned procedure. The samples were subjected to carbonization and coded by SS-B-400/500/600 (refer to carbonized temperature). After chitosan treatment, the specimens were coded by SS-BC-400/500/600 (refer to carbonized temperature) (Figure 1).

### 2.3. Adsorption Experiments

#### 2.3.1. Single-Factor Adsorption

The influence of pH, adsorption temperature, adsorption time, and adsorbent dosage on Cu(II) adsorption efficiency was methodically examined through individual factor trials. The experimental protocol involved adjusting the pH of a 100 mL Cu(II) solution (initial concentration: 30 mg L^−1^) to values of 2, 3, 4, 5, and 6 using 1 M HCl. Subsequently, 10 mg of adsorbent was introduced to each solution, followed by placement in a constant temperature shaker set at 35 °C and 150 rpm until reaching adsorption equilibrium. Post-centrifugation, the Cu(II) concentration in the supernatant was gauged using flame atomic absorption spectrometry (FAAS). For the adsorption temperature investigation, a 10 mg adsorbent quantity was combined with a 100 mL Cu(II) solution (30 mg L^−1^) under pH ≈ 5 conditions, with the mixture agitated at varying temperatures until adsorption equilibrium was attained. In the adsorption time study, at pH = 5 and 35 °C, the adsorbent was added to a Cu(II) solution of identical concentration, agitated at 150 rpm, and sampled every 10 min to determine the Cu(II) concentration via FAAS. In the adsorbent dosage experiment, conducted under conditions of solution concentration at 30 mg·L^−1^, pH = 5, and a temperature of 35 °C, the adsorbent dosage was altered to explore its impact on the adsorption efficiency (All experiments were performed in triplicate.).

#### 2.3.2. Adsorption Kinetics

A total of 100 mL of CuSO_4_·5H_2_O solution (50 mg L^−1^) was added to a conical flask, and the pH was adjusted to 5 using HCl. The flask was placed in a constant temperature water bath shaker at 35 °C for pre-equilibration. The adsorbent specimens (10 mg) were weighed and rapidly added to the solution to initiate adsorption. The samples were withdrawn at 20 min intervals. After centrifugation, the Cu(II) concentration was determined by FAAS. The adsorption capacity of Cu(II) was calculated by Equation (1) [23].

(1) $$q_{t}=\frac{\left(c_{0}-c_{t}\right)}{m}V$$

where *C*_0_ represents the initial concentration of Cu(II) (mg L^−1^); *C_t_* denotes the concentration at time *t* (mg L^−1^); *q_t_* indicates the adsorption capacity at time *t* (mg g^−1^); *V* stands for the solution volume (mL), while *m* (g) is the mass of adsorbent.

Subsequently, the experimental data were fitted using the pseudo-first-order kinetic model (Equation (2)) and the pseudo-second-order kinetic model (Equation (4)) for analysis [24].

Pseudo-first-order kinetic model formula [25]:

(2) $$q_{t}=q_{e}\left(1-e^{K_{1}t}\right)$$

(3) $$\ln\left(q_{e}-q_{t}\right)=\ln q_{e}-K_{1}t$$

Pseudo-second-order model formula [26]:

(4) $$q_{t}=\frac{K_{2}q_{e}^{2}t}{1+K_{2}q_{e}t}$$

(5) $$\frac{t}{q_{t}}=\frac{1}{K_{2}q_{e}^{2}}+\frac{t}{q_{e}}$$

where *q_e_* is the maximum saturated adsorption capacity (mg g^−1^); *q_t_* indicates the adsorption capacity at time *t* (mg g^−1^); *t* is the adsorption time (min); *K*_1_ and *K*_2_ are the rate constants for the pseudo-first-order and pseudo-second-order kinetic models, with units of min^−1^ and g mg^−1^ min.

#### 2.3.3. Adsorption Isotherm

Different concentrations of the CuSO_4_·5H_2_O solution were prepared and maintained at 35 °C, and the pH was adjusted to 5 using HCl. After pre-equilibration, the specimens were added to the flasks. After reaching the adsorption equilibrium, samples were withdrawn. Following centrifugation, the supernatant was analyzed by FAAS. The adsorption capacity was calculated using Equation (1). The experimental data were fitted to the Langmuir [27] (Equation (6)) and Freundlich (Equation (8)) isotherm models for analysis. The Langmuir model assumes a homogeneous adsorbent surface with no interactions between adsorbate molecules and monolayer coverage [28]. The Freundlich model is applicable to adsorption on heterogeneous surfaces and can describe experimental results well over a wide concentration range [29].

The Langmuir equation is as follows [30]:

(6) $$q_{e}=\frac{q_{m}K_{L}c_{e}}{1+K_{L}c_{e}}$$

(7) $$\frac{c_{e}}{q_{e}}=\frac{1}{q_{m}K_{L}}+\frac{c_{e}}{q_{m}}$$

While the Freundlich equation is as follows [31]:

(8) qe=KFce1n

(9) $$\ln q_{e}=\ln K_{F}+\frac{1}{n}\ln c_{e}$$

where *q_e_* is the equilibrium adsorption amount, mg g^−1^; *c_e_* is the Cu(II) concentration of the solution when the reaction reaches equilibrium, mg L^−1^; *q_m_* is the maximum adsorption amount, mg g^−1^; *K_L_* and *K_F_* are Langmuir and Freundlich’s adsorption affinity constants L mg^−1^, respectively, and *n* denotes the adsorption strength factor.

### 2.4. Characterization

The concentration of Cu(II) in the model aqueous solution was determined using an FAAS (ContrAA 300, Analytik Jena AG, Jena, Germany). Functional groups of the specimens were analyzed using a Fourier transform infrared (FT-IR) spectrometer (IRSpirit, Shimadzu Corporation, Kyoto, Japan) with the potassium bromide (KBr) pellet technique. The thermal stability and decomposition behavior of the specimens were characterized by a thermal analyzer (STA 449 F5, NETZSCH-Gerätebau GmbH, Selb, Germany). The surface morphology of the adsorbents was observed using a scanning electron microscope (SEM, JSM-7800F, JEOL Co. Ltd., Tokyo, Japan). The surface area, pore volume, and pore size distribution were determined via nitrogen physisorption at 77 K using an Autosorb-iQ analyzer (Quantachrome Instruments, Boynton Beach, FL, USA, model 4000e). The pore size distribution was calculated from the adsorption branch of the isotherm using the Barrett–Joyner–Halenda (BJH) model. The crystal and phase structures of the specimens were analyzed using an X-ray diffractometer (XRD) (D8 Advance, Bruker Corporation, Bremen, Germany).

## 3. Results and Discussion

### 3.1. Characterization of the Sunflower-Derived Biochar Adsorbents

**Figure 2:**
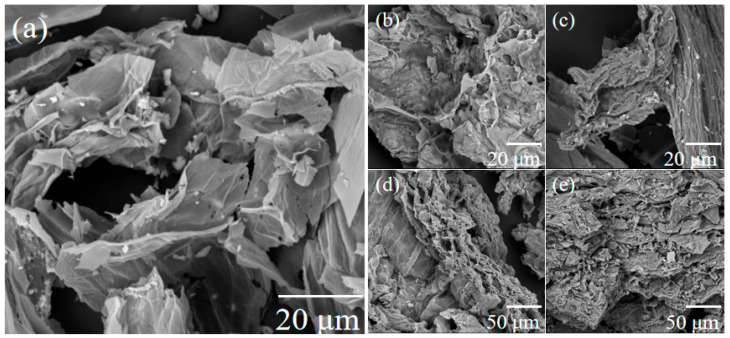
SEM images of (**a**) SS, (**b**) SS-A-500, (**c**) SS-B-500, (**d**) SS-AC-500, and (**e**) SS-BC-500.

Scanning electron microscope images of raw biochar reveal a relatively smooth surface structure and an underdeveloped pore architecture. This phenomenon primarily results from the lack of modification, extensive gasification reactions, and the formation of complex channels. SEM images of SS-A-500 and SS-B-500 are presented in Figure 2b and c, respectively. Compared with the raw SS shown in Figure 2a, rough surfaces covered with etching grooves and pits of varying depths are observed in SS-AC-500 specimens. This morphology is attributed to the strong dissolution and etching of lignin and hemicellulose fractions in raw SS by alkali treatment [32]. Figure 2c shows that the fibrous structure of SS was partially degraded after enzymatic modification, with grooves and cracks appearing on the surface, while some regions exhibit a tendency to wrinkle [33,34]. Figure 2d,e display the SEM images of SS-AC-500 and SS-BC-500 after chitosan modification of the crude biochars. Generally, the chitosan-modified alkali-treated SS-derived biochar (SS-AC-500) exhibits fragmented porous zones with a layered appearance, while the chitosan-modified biological-treated SS-derived biochar (SS-BC-500) presents a relatively fragmented and irregular flake-stacked structure.

**Figure 3:**
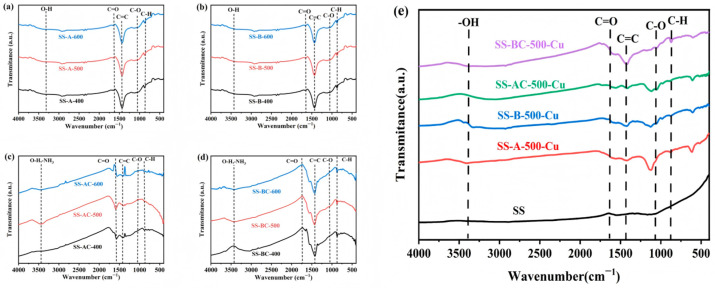
FT-IR spectrums of biochars by carbonization of (**a**) alkali and (**b**) biological treated SS under different temperatures, and the chitosan modified (**c**) alkali and (**d**) biological treated SS derived biochars, (**e**) original biomass carbon, and the four modified biochars after Cu(II) adsorption.

Functional groups on the surface of the SS-derived biochars before and after further chitosan modification are characterized by FTIR. As shown in Figure 3, for all the specimens, the absorption peaks at 876 cm^−1^, 1436 cm^−1^, and 1603 cm^−1^ are attributed to the C–H bending vibration, the C=C skeletal vibration, and the C=O stretching vibration of carboxyl groups, the predominant vibrations originate from aromatic rings [35]. The absorption peak at 1360 cm^−1^ corresponds to the C–H stretching vibration [36]. The O–H absorption peak around 3430 cm^−1^ is less pronounced, possibly due to uneven heating during carbonization of the alkali and biologically modified SS. In the specimens modified with chitosan after carbonization, an N–H absorption peak appears around 3500 cm^−1^, and a C–O stretching vibration peak is observed at 1051 cm^−1^. The lack of a distinct amino group signal is likely due to the significant overlap and masking of its characteristic absorption band around 3400 cm^−1^ by the broad and intense O-H stretching band. The FTIR spectra in Figure 3e compare the original biomass carbon with the four modified biochars post Cu(II) adsorption. The unmodified biochar shows a limited number of functional group peaks. A considerable reduction in peak intensity is observed for the modified biochars after adsorption, indicating the involvement of these functional groups in Cu(II) binding. Meanwhile, the spectra in Figure 3 also indicate that the carbonization temperatures exhibit a negligible influence on the types of functional groups.

**Figure 4:**
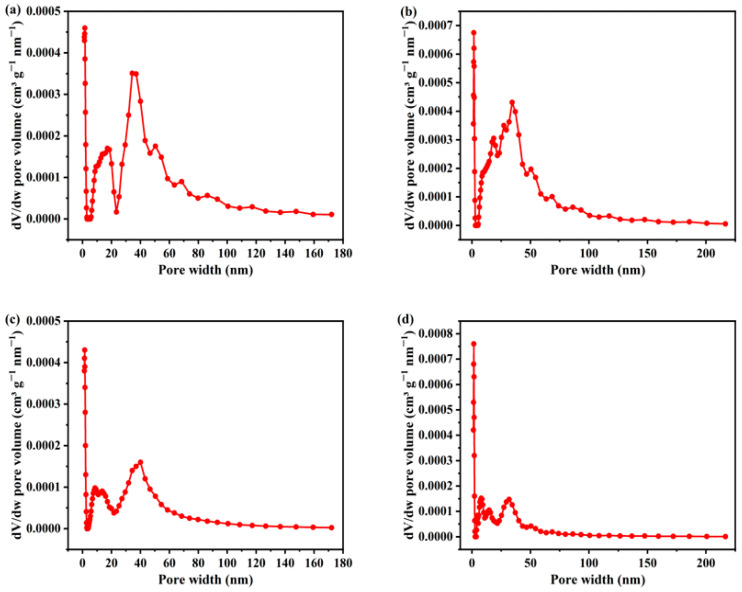
Pore size distribution (PSD) of (**a**) SS-A-500, (**b**) SS-B-500, (**c**) SS-AC-500, and (**d**) SS-BC-500.

The specific surface area measured by BET (Table 1) reveals a hierarchical evolution in porosity: SS-B-500 > SS-A-500. This trend is consistent with the morphological evolution observed via SEM. The pore size distribution (PSD) curves presented in Figure 4a,b demonstrate that SS-A-500 exhibits a highly concentrated, narrow distribution of mesopores (primary peak centered at 20–40 nm), indicative of strong pore structure homogeneity. In contrast, SS-B-500 is dominated by broadly distributed mesopores/macropores (primary peak spanning 50–150 nm), covering a wide pore size range and accompanied by a minor fraction of small mesopores. Figure 4c,d presents the chitosan-modified biochar sample (SS-AC/BC-500). The results clearly show a decrease in both the total pore volume and specific surface area of the modified biochar. This reduction occurs because chitosan molecules enter the larger pores (particularly mesopores and macropores), adhere to the pore walls, and in some cases, block the channels entirely. Consequently, the pore volume of mesopores and macropores decreases significantly.

**Figure 5:**
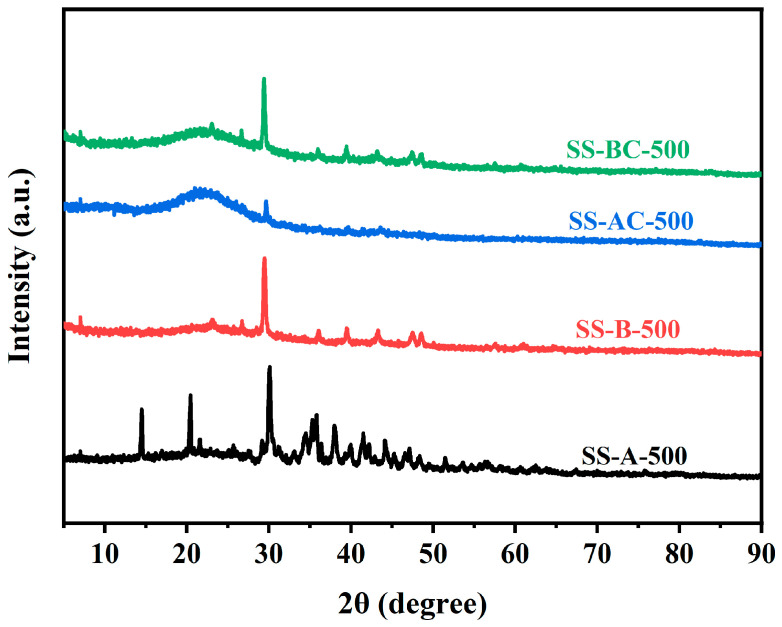
XRD patterns of SS-A-500, SS-B-500, SS-AC-500, and SS-BC-500.

Figure 5 presents the XRD patterns of SS-A-500, SS-B-500, SS-AC-500, and SS-BC-500. All the tested specimens exhibit an almost amorphous phase with unique properties. Specifically, the characteristic cellulose doublet within the ranges of 15–16° and 22–25° is observed, consistent with the typical crystalline features of natural plant fibers [37]. The structures of the tested specimens are similar, predominantly exhibiting an amorphous character [32]. The appearance of the 002 peak (2*θ* = 22–25°), attributed to the parallel stacking of aromatic layers [38], indicates the parallelism and stacking height of aromatic sheets. The XRD patterns also demonstrate that chitosan modification significantly impacts cellulose crystallinity. The crystallinity increases in the specimens derived from alkali-treated SS, whereas it decreases in the specimens derived from biologically treated SS due to structural degradation [39]. The predominant amorphous nature is mainly due to the porous structure of the biochars.

**Figure 6:**
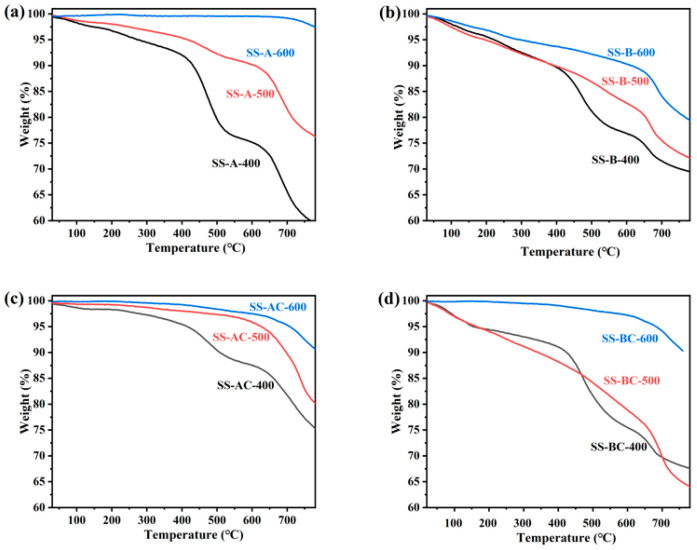
TG curves of various SS-derived biochar adsorbents (**a**) alkali and (**b**) biological treated SS under different temperatures, and the chitosan-modified (**c**) alkali and (**d**) biological treated SS-derived biochars.

The thermochemical properties of the biochar adsorbents derived from SS are investigated via TGA. The TG curves of SS-A-400 and SS-A-500 indicate that the specimens undergo partial decomposition in the range of 0–100 °C (Figure 6a). Due to uneven heating, two fluctuations are observed around 500 °C and 650 °C. In Figure 6b, decomposition commences at the onset of heating, which was possibly due to the presence of inorganic constituents in SS-B-400/500/600. In addition, slight decomposition occurs between 0 and 200 °C. SS-AC-500 and SS-AC-600 begin significant weight loss/decomposition around 450 °C, with total weight losses reaching approximately 80% and 90%, respectively (Figure 6c). The lower weight loss of SS-AC-600 indicates its relatively better thermal stability. Moreover, partial decomposition of the specimens occurred immediately upon heating for SS-BC-400 and SS-BC-500, suggesting the possible presence of impurities or inorganic components (total weight losses reaching about 70%) (Figure 6d). In contrast, SS-BC-600 started decomposing at 400 °C and exhibits a higher weight loss of approximately 90% at ~780 °C. The decomposition behavior of SS-BC-600 suggests this group exhibits superior thermal stability compared to other specimens.

### 3.2. Analysis of Single-Factors

**Figure 7:**
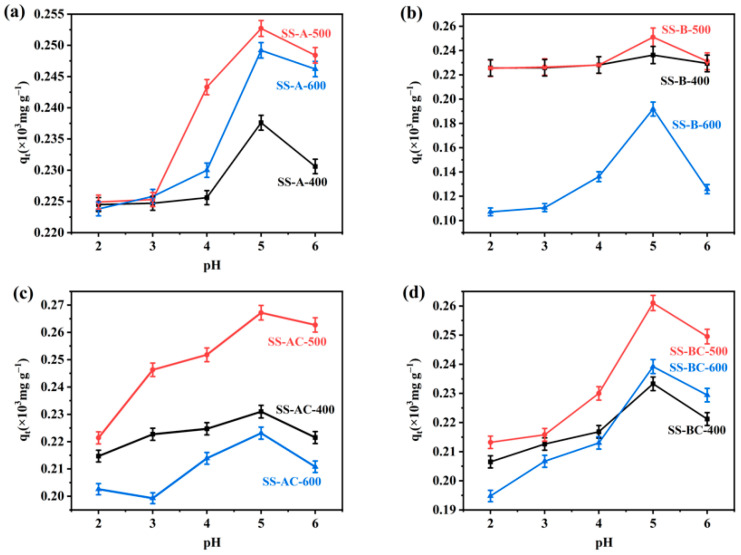
Adsorption efficiency curves of Cu(II) under varying pH conditions for biochars produced by carbonization of (**a**) alkali and (**b**) biological treated SS at different temperatures, and the chitosan-modified (**c**) alkali and (**d**) biological treated SS-derived biochars. The amount of adsorbent used was 0.01 g, and the initial concentration of Cu(II) was 30 mg L^−1^, mean ± SD (*n* = 3).

The good physio-chemical performances and the good thermostability of the candidate SS-derived adsorbents led us to further investigate the Cu(II) adsorption performances of the specimens from model aqueous solutions. As the pH value significantly influences the metal ions adsorption performance by affecting the functional groups on the surface of different adsorbent materials [40,41]. At first, the effect of pH on Cu(II) adsorption by the SS-derived materials was evaluated. Figure 7 shows the adsorption efficiency curves for Cu(II) under varying pH conditions. At pH < 4, the Cu(II) adsorption efficiency by the SS-A/B-400/500/600 specimen decreased because hydrogen ions (H^+^) and copper ions (Cu(II)) competed for binding sites on the surface of materials. As the pH increased, both SS-A-500 (Figure 7a) and SS-B-500 (Figure 7b) reached the maximum adsorption efficiency at pH 5. This phenomenon can be attributed to the increased availability of functional groups on the adsorbent surface for binding with Cu(II).

After modification with chitosan, the Cu(II) removal efficiency for SS-AC-500 and SS-BC-500 specimens was enhanced (Figure 7c,d), which is related to the intrinsic structure of chitosan. During adsorption, Cu(II) was first attracted to the surface of the chitosan-modified biochars, followed by interactions with functional groups (e.g., amino and hydroxyl groups) in the chitosan molecules, thereby enhancing adsorption capacity [42]. However, lower pH values can negatively impact the functional groups of the chitosan-modified specimens. Amino groups (-NH_2_) and hydroxyl groups (-OH) were more readily protonated under acidic conditions. The protonated amino and hydroxyl groups carry a positive charge, which weakened the electrostatic interactions between the adsorbents and the positively charged Cu(II) [43]. Conversely, the pH increase reduced the number of positive charges on the surface [44], facilitating the formation of ring-shaped chelates between Cu(II) and the amino/hydroxyl groups.

**Figure 8:**
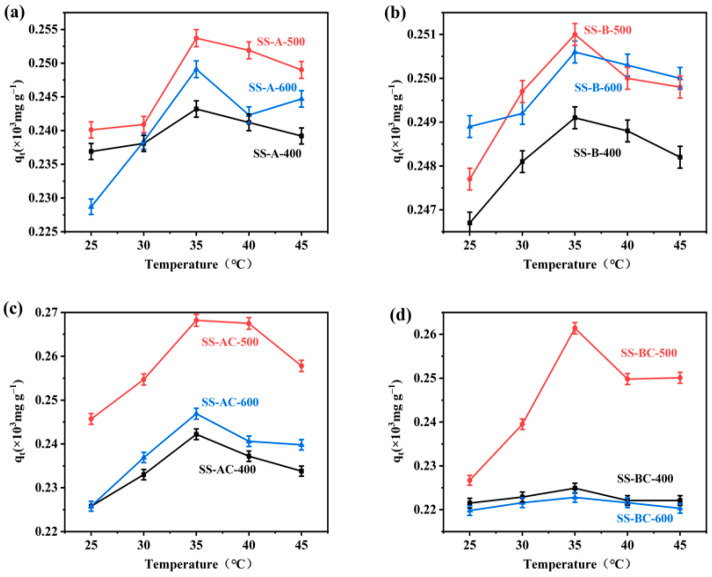
Adsorption capacity curves of Cu(II) for biochars produced by carbonization of (**a**) alkali and (**b**) biological treated SS at different temperatures, and the chitosan-modified (**c**) alkali and (**d**) biological treated SS-derived biochars. The amount of adsorbent used was 0.01 g, and the initial concentration of Cu(II) was 30 mg L^−1^, mean ± SD (*n* = 3).

The adsorption temperature also showed a significant influence on the metal ions separated from the aqueous solutions [45]. Figure 8 presents the adsorption capacity curves for Cu(II) at different temperatures. As expected, temperature exerted an obvious influence on the Cu(II) adsorption, with the optimized adsorption capacity at 35 °C for all specimens. As shown in Figure 8, the Cu(II) adsorption increased gradually in the tested groups as the temperature rose from 20 °C to 35 °C. This enhancement can be attributed to the endothermic nature of the adsorption process (ΔH > 0) [46]. Specifically, Figure 8a indicates that the adsorption capacity of alkali-modified biochar (SS-A-500) reached the maximum of 253.7 mg g^−1^ adoptability of Cu(II) at 35 °C. Figure 8b indicates that the enzymatically modified biochar (SS-B-500) also achieved its highest adsorption capacity of 251 mg g^−1^ at the same temperature. The adsorption performance of some chitosan-modified biochars is inferior to that of alkali-modified biochars. This phenomenon may be attributed to the blockage of partial pores in biochars by chitosan, which further leads to a decrease in specific surface area and ultimately results in the reduction of the adsorption capacity.

According to Figure 8c,d, the Cu(II) adsorption capacities increased after chitosan modification, in contrast to the corresponding biochars without chitosan modification, which clearly shows the synergistic effect for the Cu(II) adsorption by two-stage material modification before and after carbonization. This improvement is attributed to interactions between functional groups in chitosan molecules and Cu(II), which provided additional adsorption sites [47]. However, as the temperature increased beyond 35 °C, the Cu(II) adsorption exhibited a significant declining trend for SS-AC/BC-500 specimens. On the one hand, the elevated temperature may reduce the adsorption stability. On the other hand, temperature may alter the stability of chitosan [48], which is unfavorable for Cu(II) adsorption. Therefore, the adsorption of Cu(II) by SS-derived adsorbent was an endothermic process, and 35 °C was identified as the optimal adsorption temperature for both the biochars and the chitosan-modified specimens, no matter the modification protocols.

**Figure 9:**
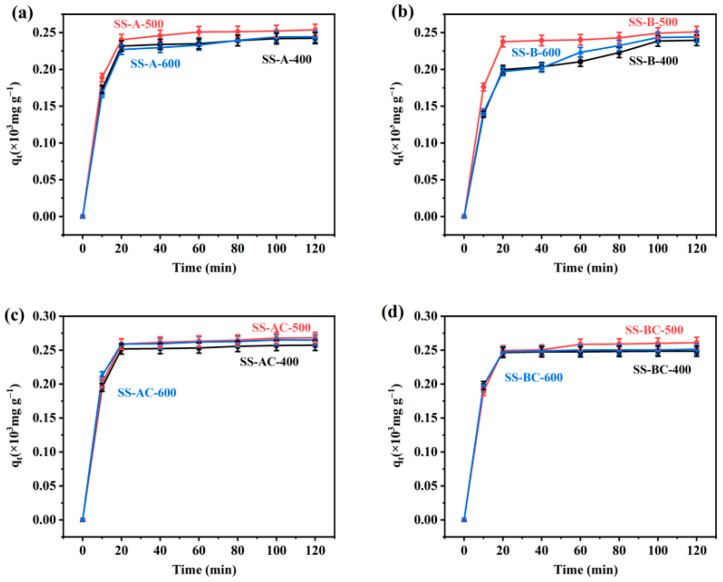
Cu(II) adsorption capacity at different contact times on (**a**,**b**) SS-derived biochars carbonized at different temperatures and (**c**,**d**) their chitosan-modified derivatives. The amount of adsorbent used was 0.01 g, and the initial concentration of Cu(II) was 30 mg L^−1^, mean ± SD (*n* = 3).

To determine the adsorption equilibrium time, the Cu(II) removal efficiency by different SS-derived materials was investigated as a function of contact time. As shown in Figure 9, during the adsorption of Cu(II), the adsorption capacity for all candidate materials increased rapidly in the initial stage. As the adsorption process proceeded, the adsorption capacity approached the maximum value within 20 min. This rapid rate at the initial stage can be attributed to the abundance of available adsorption sites on the surface of specimens, allowing easy access and binding of Cu(II) [49]. However, with further increases in the contact time (until equilibrium was reached), the Cu(II) removal efficiency did not significantly increase, because the active sites on the adsorbent became saturated and were unable to accommodate further adsorption with the lengthening of the adsorption time.

**Figure 10:**
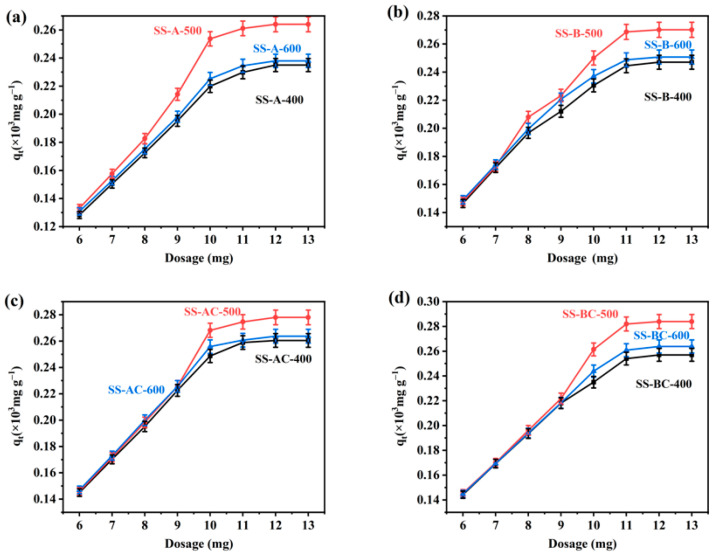
Adsorption efficiency of Cu(II) at different adsorbent dosages for biochars from (**a**) alkali and (**b**) biological-treated SS (carbonized at different temperatures) and their chitosan-modified derivatives (**c**,**d**). The initial concentration of Cu(II) was 30 mg L^−1^, mean ± SD (*n* = 3).

The adsorbent dosage determines the adsorption capability for a metal ion-containing solution of specific initial concentration [50]. The effect of adsorbent dosage on the adsorption capacity of Cu(II) is presented in Figure 10. The results indicate that the adsorption percentage of Cu(II) initially increased with the increase in adsorbent dosage. This enhancement was attributed to the higher availability of active sites at higher adsorbent dosages, leading to increased adsorption of Cu(II) as the biochar concentration increases [51]. However, after the available Cu(II) ions were largely adsorbed, further increases in adsorbent dosage exerted a diminishing effect on adsorption.

### 3.3. Adsorption Kinetics Fitting Analysis

**Figure 11:**
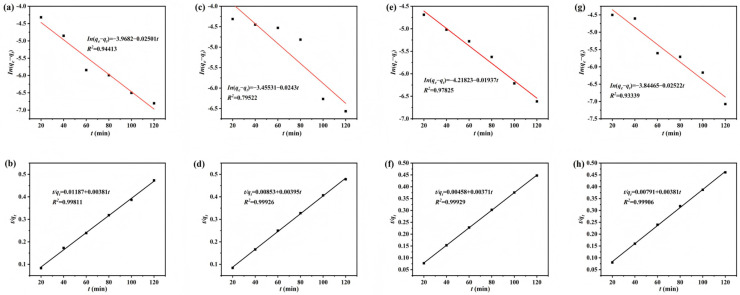
Linear fitting plots of the pseudo-first-order and pseudo-second-order kinetic models for (**a**,**b**) alkali and (**c**,**d**) biological, and the chitosan-modified (**e**,**f**) alkali and (**g**,**h**) biologically treated SS-derived biochars.

To investigate the rate and mechanism of the Cu(II) adsorption, the adsorption kinetics of the candidate SS-derived materials were further analyzed. Figure 11 displayed the linear fitting of the pseudo-first-order and pseudo-second-order kinetic models for SS-A-500, SS-B-500, SS-AC-500, and SS-BC-500, respectively. The coefficients of determination (*R*^2^) for the pseudo-second-order model were consistently higher than those for the pseudo-first-order model across the four specimens. This result suggested that the Cu(II) adsorption on the SS-derived materials was predominantly governed by chemical adsorption. Successful multi-stage modification of the SS precursor introduced abundant active functional groups, such as hydroxyl and amino groups, into the materials’ structure. These functional groups complex with Cu(II) and thereby facilitate the adsorption process. The rapid adsorption kinetics indicated that the Cu(II) adsorption relies on the availability of open binding sites on the specimens.

### 3.4. Adsorption Isotherm Fitting Analysis

**Figure 12:**
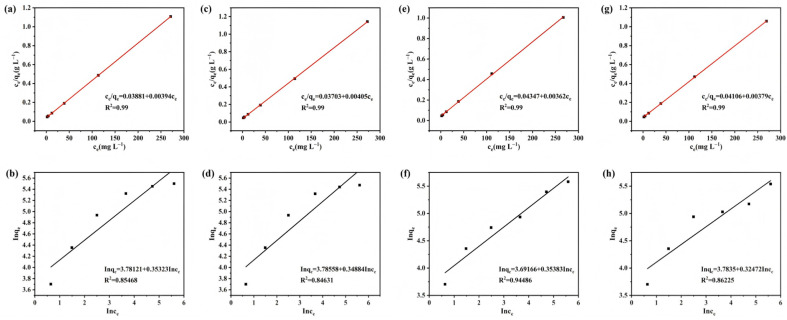
Langmuir and Freundlich fitting curves for Cu(II) adsorption on (**a**,**b**) SS-A-500, (**c**,**d**) SS-B-500, (**e**,**f**) SS-AC-500, and (**g**,**h**) SS-BC-500.

Figure 12 presents the Langmuir and Freundlich fitting plots for SS-AC-500 and SS-BC-500. The Langmuir model was based on specific assumptions, including the presence of identical adsorption sites on the adsorbent surface and the formation of only a monolayer coverage of adsorbate molecules [52]. The Freundlich model assumed a uniform distribution of adsorbate molecules on the adsorbent surface [53].

In this study, adsorption isotherm fitting was employed to investigate the maximum saturated adsorption capacity. The adsorption capacity increased with rising Cu(II) concentration in model solutions as the adsorption sites became progressively occupied. Figure 12a,b show the Langmuir and Freundlich fitting curves, respectively, for Cu(II) adsorption by SS-AC-500. It can be observed that the coefficient of determination (*R*^2^) for the Langmuir fit was higher than that for the Freundlich fit. Similarly, Figure 12c,d display the Langmuir and Freundlich fitting curves for Cu(II) adsorption by SS-BC-500, with the Langmuir model exhibiting a higher *R*^2^ value than the Freundlich model. These results indicate that the Langmuir model provides a better fit for Cu(II) adsorption by SS-AC-500 and SS-BC-500 specimens. Therefore, the adsorption of Cu(II) onto SS-AC-500 and SS-BC-500 was predominantly governed by monolayer adsorption. Table 2 shows the parameters of the Langmuir and Freundlich models.

### 3.5. Analysis of Regenerative Effects and Comparison Study

**Figure 13:**
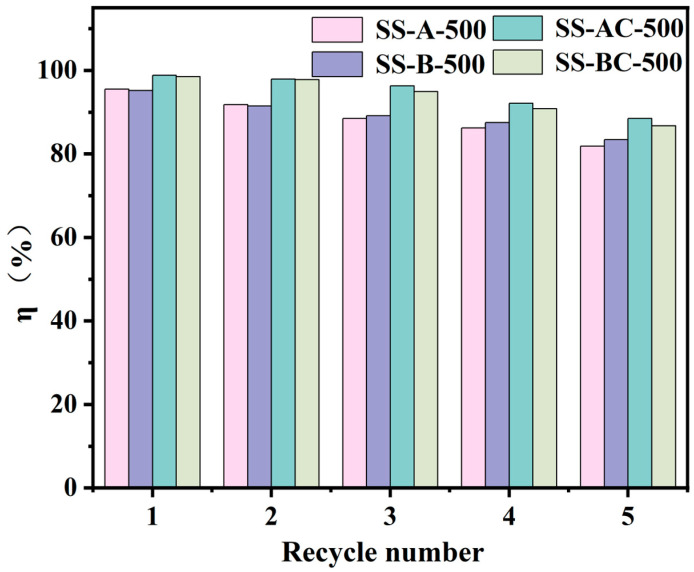
Desorption and regeneration effects of SS-A-500, SS-B-500, SS-AC-500, and SS-BC-500.

The adsorbents’ (SS-A-500, SS-B-500, SS-AC-500, and SS-BC-500) regenerability and reusability were assessed through five consecutive adsorption–desorption cycles, as depicted in Figure 13. The Cu(II) removal efficiency of all samples declined gradually with increasing cycle numbers, primarily due to the loss of active sites and mass during regeneration. Despite this, even after five cycles, all modified samples exhibited strong performance, maintaining removal rates above 80%. SS-A-500, SS-B-500, SS-AC-500, and SS-BC-500 achieved final removal efficiencies of 81.85%, 83.40%, 88.45%, and 86.75%, respectively. Notably, SS-AC-500 and SS-BC-500 displayed enhanced stability and less performance decay compared to SS-A-500 and SS-B-500. These findings unequivocally highlight the exceptional reusability and structural stability of the modified adsorbent, positioning it as a promising material for practical wastewater treatment applications.

Table 3 presents the Cu(II) adsorption capacities of various biochar types. The comparative results indicate the adsorption capacities of biochar derived from different biomass feedstocks, produced at varying pyrolysis temperatures, and subjected to distinct modification conditions. The adsorbent developed in this work exhibits a maximum adsorption capacity (q_max_) for Cu(II) that surpasses most previously reported adsorbents. Moreover, it requires a notably lower carbonization temperature during synthesis compared to other adsorbent types. This combination of high uptake capacity and low energy input for preparation underscores its potential for practical wastewater remediation, enabling efficient heavy metal removal with reduced treatment costs. In this study, sunflower straw biochar (SS) demonstrated significant adsorption potential. Following alkali modification (SS-A-500) and enzyme modification (SS-B-500), the adsorption performance of the modified chitosan (SS-AC/BC-500) was further enhanced.

### 3.6. Sorption Mechanisms

**Figure 14:**
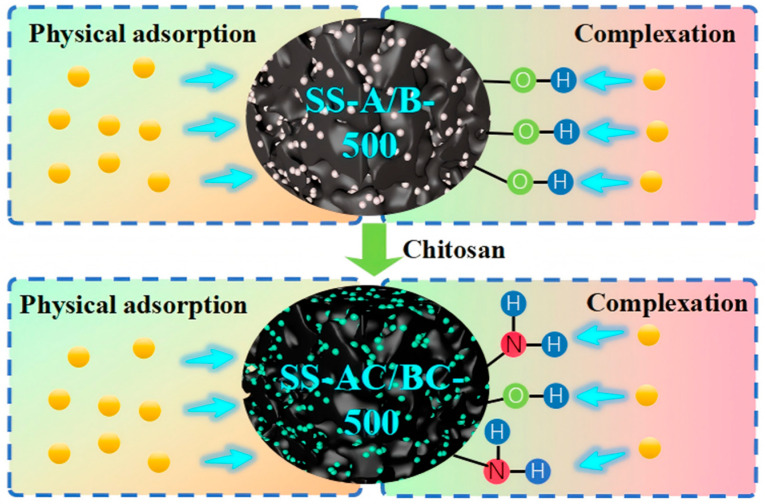
Cu(II) adsorption mechanism.

Based on the results of material characterization and single-factor adsorption experiments, this study systematically summarizes the enhancement mechanism of Cu(II) adsorption by SS-biochar. The introduction of chitosan resulted in a strong fit to both the Langmuir isotherm model and the pseudo-second-order kinetic model, indicating that the adsorption process adheres to a monolayer chemical adsorption mechanism. This finding indirectly confirms the occurrence of specific coordination reactions between functional groups, such as amino groups, and Cu(II). Experiments examining the influence of solution pH further demonstrated that, under weakly acidic conditions, deprotonated amino groups (-NH_2_) are more likely to serve as the primary adsorption sites for Cu(II). In summary, we conclude that in this adsorption system, the amino group plays a dominant role, while the hydroxyl group contributes a synergistic coordination effect. Figure 14 illustrates the adsorption mechanism of Cu(II) by SS-A/B-500 and SS-AC/BC-500.

## 4. Conclusions

This study provides a novel method for the effective Cu(II) adsorbent derived from SS by sequential modification of the lignocellulosic template and the carbonized skeleton. The effects of carbonization temperature (400–600 °C) and surface modification strategies (NaOH and enzyme treatment before carbonization, and chitosan modification post-carbonization) on material characteristics and the Cu(II) adsorption performance were investigated. FT-IR, XRD, and TG analyses confirmed that the SS-derived biochars possessed abundant functional groups (e.g., -COOH, -OH) on the surface and good thermal stability. Among the candidate specimens, the sequential cellulase–carbonization–chitosan co-modified material (SS-AC-500) exhibited the maximum Cu(II) adsorption capacity (268.2 mg g^−1^) under the conditions of 10 mg of adsorbent dosage, pH 5, and 35 °C for 120 min adsorption. Meanwhile, the adsorption process followed the pseudo-second-order kinetic model (*R*^2^ > 0.99). The Langmuir isotherm model indicated that the SS-AC-500 exhibited the highest correlation coefficient (*R*^2^ > 0.99), suggesting the Cu(II) adsorption was primarily homogeneous monolayer adsorption. The recycled materials could be obtained at about an 80% recovery level of desorbing metal ions after five cycles. These high-performance, low-cost, and environmentally friendly SS-derived adsorbents by sequential modification exhibit considerable potential for remediating heavy metal-contaminated wastewater.

## Figures and Tables

**Table 1:** Textural properties from BET analysis (mean ± SD, *n* = 2).

Adsorbents	BET Surface Area
SS-A-500	147 ± 5 m^2^ g^−1^
SS-B-500	162 ± 5 m^2^ g^−1^
SS-AC-500	103 ± 4 m^2^ g^−1^
SS-BC-500	119 ± 4 m^2^ g^−1^

**Table 2:** Parameters of Langmuir and Freundlich for Cu(II) adsorption using biochar.

Adsorbent			Langmuir			Freundlich	
	qe cal (mg g^−1^)	qe exp (mg g^−1^)	K_L_	R^2^	1/n	K_f_	R^2^
SS-A-500	253.7	254.2	0.0996	0.99	0.584	10.35	0.85
SS-A-500	251.0	251.8	0.1002	0.99	0.578	11.28	0.84
SS-AC-500	268.2	268.9	0.0945	0.99	0.462	18.52	0.94
SS-BC-500	261.4	262.3	0.0989	0.99	0.472	19.51	0.86

**Table 3:** Comparison of Cu(II) adsorption capacity by different types of biochar.

Type of Biochar	Pyrolysis Temperature (°C)	Modifying Agent	Adsorption Capacity (mg g^−1^)	Reference
Coconut shells	600	Calcite	213.9	[54]
Shrimp shell	800	NaOH	141.76	[55]
Sesame	600	Aniline	124.78	[56]
Coffee Ground	800	KOH	75.02	[57]
Brown algae	700	Ethylenediamine	105.3	[58]
SS-A-500	500	NaOH	253.7	Present study
SS-B-500	500	Cellulase	251.0	Present study
SS-AC-500	500	NaOH + Chitosan	268.2	Present study
SS-BC-500	500	Cellulase + Chitosan	261.4	Present study

## Data Availability

The original contributions presented in this study are included in the article. Further inquiries can be directed to the corresponding authors.
